# Concordance between initial GeneXpert MTB/RIF results and follow-up diagnostic testing in patients treated for multidrug-resistant tuberculosis in Lusaka, Zambia

**DOI:** 10.11604/pamj.2025.52.33.43309

**Published:** 2025-09-22

**Authors:** Thijs Hoffman, Maurice Mwansa, Angel Mubanga, John Kondwelani Mateyo

**Affiliations:** 1 Department of Internal Medicine, University Teaching Hospital, Lusaka, Zambia,; 2 Department of Pulmonology, St. Antonius Hospital, Nieuwegein/Utrecht, The Netherlands,; 3 National Tuberculosis and Leprosy Control Program, Lusaka, Zambia

**Keywords:** Rifampicin resistance, false positive, multidrug-resistant tuberculosis, tuberculosis

## Abstract

**Introduction:**

diagnosis of multi-drug-resistant (MDR) tuberculosis (TB) has been revolutionized by the introduction of the GeneXpert MTB/RIF test, which is much faster and easier to use than traditional methods. Concerns have been raised about false positive results for rifampicin susceptibility, especially in cases of samples with low bacillary loads and settings with a low burden of MDR-TB. The objective of this study was to assess the concordance between initial GeneXpert MTB/RIF results and subsequent diagnostic testing for rifampicin susceptibility in patients with suspected MDR-TB in Lusaka, Zambia.

**Methods:**

this was a retrospective cohort study of patients who started MDR-TB treatment in Lusaka province, Zambia, from 2019 to 2021. Initial GeneXpert MTB/RIF bacillary load and rifampicin susceptibility, as well as results for any confirmatory testing on rifampicin resistance (including repeat GeneXpert MTB/RIF, phenotypic drug susceptibility testing, and line probe assay results) and patient outcomes were collected from patient files.

**Results:**

the study included 342 patients. Initiation of MDR-TB treatment was based on rifampicin resistance detected by GeneXpert MTB/RIF in all patients. Twenty-nine percent of patients had a very low bacillary load in the initial sample. Additional diagnostics for rifampicin resistance were available in 56% of patients, and revealed rifampicin susceptibility in 28% of tested patients. Rifampicin-susceptible TB was found more often if the bacillary load in the initial sample was very low (78% versus 18%; p=0.0001).

**Conclusion:**

false positive results for rifampicin susceptibility on GeneXpert MTB/RIF appear to be common in Lusaka, Zambia, especially in patients with very low bacillary loads on the initial test. Repeat testing to confirm MDR-TB prior to initiation of treatment can be considered in this group.

## Introduction

Tuberculosis (TB) remains an important global health problem, which is especially pressing in sub-Saharan Africa [1]. *Mycobacterium tuberculosis* that is resistant to the two most effective drugs against TB, rifampicin and isoniazid, is termed multi-drug-resistant (MDR)-TB [2]. MDR-TB is increasingly recognized to be a threat to efforts to achieve global TB control [2]. The treatment of MDR-TB is longer compared to standard TB treatment, and has less favourable outcomes [2].

Formally, resistance to both rifampicin and isoniazid is required for the diagnosis of MDR-TB. However, because most isolates that are resistant to rifampicin are also resistant to isoniazid, rifampicin-resistant tuberculosis is routinely treated as MDR-TB [2]. Historically, diagnosing MDR-TB has been challenging, as results from phenotypic drug susceptibility testing (DST) on culture isolates can take weeks or months to become available. Recently, molecular testing methods have become available, including line probe assay (LPA) and GeneXpert. The widespread introduction of the cartridge-based GeneXpert platform in endemic countries has greatly expedited the diagnosis of MDR-TB [3]. GeneXpert MTB/RIF simultaneously tests for the presence of *Mycobacterium tuberculosis* in a sample, and quantifies this into one of four categories: high, medium, low, or very low. Furthermore, the assay can detect mutations within the rpoB gene region that confer resistance to rifampicin. Results from the assay are usually available within two hours.

Some reports have raised concerns about false positive results for rifampicin resistance, as detected by GeneXpert MTB/RIF, especially in cases of a low bacillary load and in a setting of low MDR-TB prevalence [4-8]. In Zambia, about 50,000 new cases of TB were diagnosed in 2021, and 100% were tested with GeneXpert MTB/RIF or other rapid molecular diagnostics [3]. The burden of MDR-TB in Zambia is estimated to be 3% of all TB, which is relatively low in comparison to other countries [3]. Whether false positive rifampicin resistance is also a concern in Zambia, and to what degree, has not been studied extensively.

## Methods

**Study design and setting:** this was a retrospective cohort study that included all patients who initiated MDR-TB treatment through the MDR-TB program at the University Teaching Hospital in Lusaka, Zambia, from 2019 to 2021 (purposive sampling). This program serves all patients with MDR-TB in Lusaka province.

**Research questions:** research questions for the study were: (1) in patients diagnosed with MDR-TB based on GeneXpert MTB/RIF in Lusaka, Zambia, what follow-up diagnostic testing is available, and how often does this confirm MDR-TB? (2) Is the sputum bacillary load from the initial sample related to false-positive GeneXpert MTB/RIF rifampicin resistance? (3) Is false-positive GeneXpert MTB/RIF rifampicin resistance related to patient outcomes?

**Figure 1:**
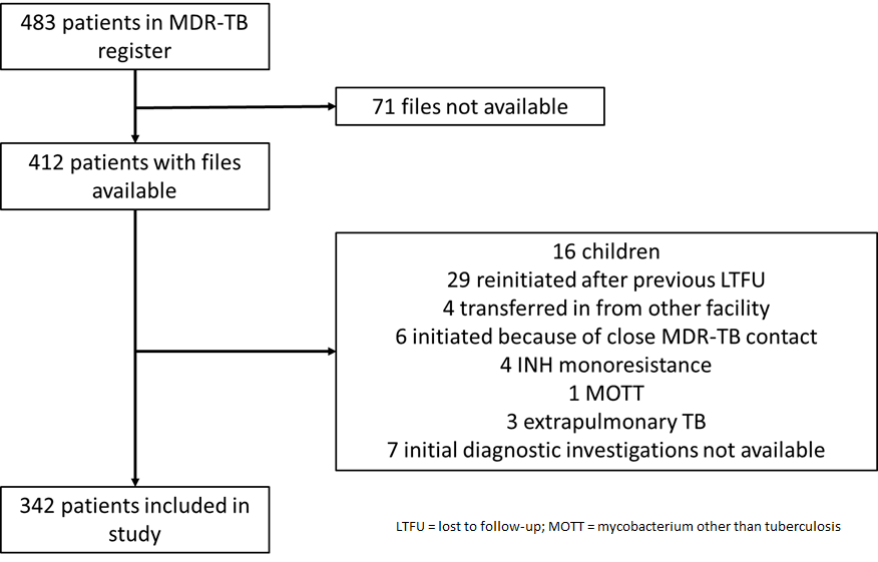
study flow chart

**Study participants:** an overview of study inclusion is provided in Figure 1. The MDR-TB register for the study period contained 483 patients. For 71 patients, files were not available for review, and 70 patients were excluded from the study for various other reasons.

**Variables and data sources:** data were extracted by the investigators from patient records, as well as from the hospital laboratory system. Collected data included: patient demographics, initial diagnostic test results, reason for starting MDR-TB treatment, the chosen MDR-TB treatment regimen, any follow-up diagnostic testing for rifampicin resistance, and treatment outcomes. Patient outcomes were followed up to September 2022.

In accordance with local guidelines, diagnostic tests for confirmation of MDR-TB are requested if patients have rifampicin resistance as determined by GeneXpert MTB/RIF [9]. This included sputum LPA, mycobacterial culture, and phenotypic DST. While these results are pending, MDR-TB treatment is initiated in accordance with the guidelines [9]. Sputum smear microscopy was used for monitoring treatment response [9].

GeneXpert MTB/RIF has been used as a first-line test for the diagnosis of TB in Zambia since 2017. Presence or absence of *Mycobacterium tuberculosis* in a sample is reported as either MTB detected-high, MTB detected-medium, MTB detected-low, MTB detected-very low, or MTB not detected. In patients where it was only reported in the file that GeneXpert MTB/RIF was positive, and where original results were not available, this was classified as level not reported. The presence or absence of rifampicin resistance is reported as detected, indeterminate, or not detected.

Line probe assay (LPA) for determining resistance to first-line TB drugs (rifampicin and isoniazid) and second-line TB drugs (fluoroquinolones and injectables) was done using the GenoTypeMTBDRsl assay (Hain Life Science GmbH, Nehren, Germany). Phenotypic DST for first-line TB drugs (rifampicin, isoniazid, streptomycin, and ethambutol) was performed using the BACTEC™ 960 Mycobacteria Growth Indicator Tube (MGIT™) system (Becton, Dickinson and Company, Franklin Lakes, NJ, USA). The *M. tuberculosis* H37Rv strain was routinely utilized as a control strain.

**Data management and statistical analysis:** the primary outcome of this study is the percentage of patients who have rifampicin resistance confirmed by follow-up diagnostic testing (i.e. repeat GeneXpert MTB/RIF, LPA, and/or phenotypic DST), stratified by four categories of bacillary load of the initial GeneXpert MTB/RIF test: high, medium, low, and very low. Percentages of confirmed rifampicin resistance per bacillary load category are compared using a Chi-square test. Secondary outcomes are the relation between confirmed positive rifampicin resistance results and patient demographics, as well as treatment outcomes. Analyses were performed using IBM SPSS Statistics for Windows, version 26 (IBM Corp., Armonk, N.Y., USA). P-values of <0.05 were considered to represent statistical significance.

**Ethical approval:** approval to conduct this study was granted by the University of Zambia Biomedical Research Ethics Committee (UNZABREC) under reference number 3958-2023. A waiver was given for patient-informed consent.

## Results

Three hundred forty-two patients were included in the study. Patient characteristics are provided in Table 1. Sixty-nine percent of the patients were male, and the median age was 34.5 years (interquartile range 28.0-43.3). Fifty-eight percent of patients were infected with the human immunodeficiency virus (HIV). All patients were started on MDR-TB treatment because of the detection of rifampicin resistance with GeneXpert MTB/RIF. The bacillary load was reported as high, medium, low, and very low in 16%, 21%, 21%, and 29% of patients, respectively. In 14% of patients, a positive GeneXpert was reported, but the bacillary load was not reported. Eighty-six percent of patients initiated treatment as outpatients.

**Table 1:** baseline characteristics of 342 patients treated for multidrug-resistant tuberculosis

Males (%)	236 (69)
Females (%)	106 (31)
Median age, years (interquartile range)	34.5 (28.0-43.3)
HIV-positive (%)	199 (58)
Previous TB* (%)	140 (42)
Respiratory symptoms at presentation# (%)	291 (93)
Constitutional symptoms at presentation# (%)	183 (59)
Duration of symptoms >2 weeks$ (%)	147 (76)
Chest X-ray available (%)	82 (24)
Showing abnormalities (%)	80 (98)
**Initial sputum appearance**	
Mucoid (%)	121 (80)
Salivary (%)	29 (19)
Blood-stained (%)	1 (1)
**Initial sputum acid-fast bacilli**	
Positive (%)	13 (50)
Negative (%)	13 (50)
**Initial sputum GeneXpert**	
MTB detected high (%)	55 (16)
MTB detected medium (%)	70 (20)
MTB detected low (%)	71 (21)
MTB detected very low (%)	99 (29)
MTB detected, level not reported (%)	47 (14)
**Initial sputum GeneXpert MTB/RIF**	
Positive (%)	342 (100)
**Reason for initiating MDR-TB treatment**	
Rifampicin resistance detected with sputum GeneXpert	342 (100)
Treatment setting at initiation>	
Inpatient (%)	48 (14)
Outpatient (%)	292 (86)

* : not available for 6 patients; #: not available for 30 patients; $: not available for 148 patients; >: not available for 2 patients

**Follow-up diagnostic testing for rifampicin resistance:** results for one or more follow-up tests for rifampicin resistance done at the time of initiation of MDR-TB treatment were available in 191 patients (56%). This included a repeat GeneXpert MTB/RIF in 163 patients, LPA in 53 patients, and phenotypic DST in 111 patients. In 30 patients, LPA or phenotypic DST was performed after initiation of MDR-TB treatment.

In patients for whom no results for follow-up testing for rifampicin resistance were available at the time of initiation of treatment for MDR-TB, repeat testing confirmed TB in 11 patients, did not confirm TB in 62 patients, and was not available at all in 78 patients.

Follow-up tests confirmed rifampicin resistance in 138 patients (72% of patients with follow-up testing available). Rifampicin susceptibility was detected in 28% of tested patients, and was identified by repeat GeneXpert MTB/RIF in 28 patients, LPA in 12 patients, phenotypic DST in a sample taken prior to treatment inititation in 33 patients, and phenotypic DST in a sample taken after initiation of MDR-TB treatment in 7 patients.

**Figure 2:**
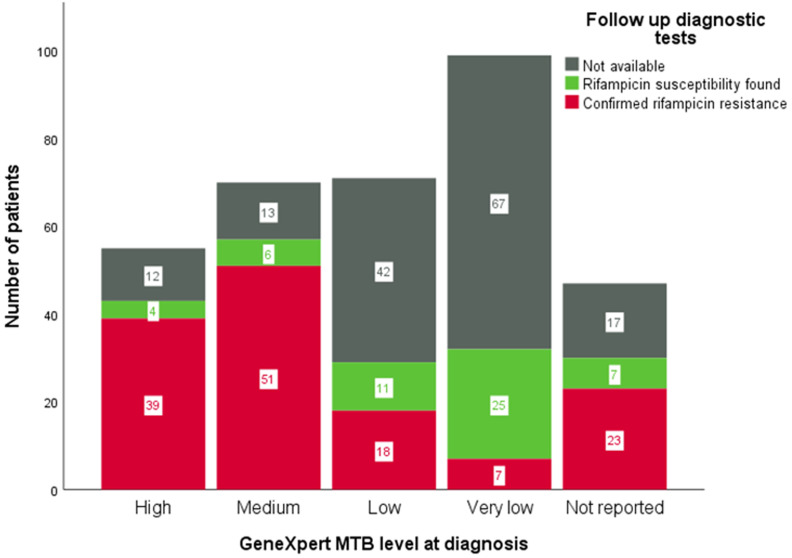
results of follow-up tuberculosis diagnostics stratified by initial GeneXpert MTB/RIF level

**Relation between bacillary load and false positive rifampicin resistance on GeneXpert MTB/RIF:** of 99 patients who had a bacillary load that was categorized as very low, rifampicin resistance was confirmed in 7 (7%). This is equal to 22% of patients with very low bacillary load in whom additional testing for rifampicin resistance was done, compared to 82% of patients with other bacillary loads in whom additional testing for rifampicin resistance was done (p=0.0001, Chi-square test) (Table 2, Figure 2). Rifampicin susceptibility was detected in 25 patients with very low bacillary load on the initial GeneXpert MTB/RIF. This was done by repeat GeneXpert MTB/RIF in 16 patients, phenotypic DST in 15 patients, LPA in 6 patients, and phenotypic DST after initiation of MDR-TB treatment in 1 patient.

**Table 2:** results of follow-up tuberculosis diagnostics stratified by initial GeneXpert MTB/RIF level (P-values calculated using Chi-square test)

Outcome	High (N=55)	Medium (N=70)	Low (N=71)	Very low (N=99)	Level not reported (n=47)	Total (N=342)
Rifampicin resistance confirmed with additional diagnostics (%)	39 (71)	51 (73)	18 (25)	7 (7)	23 (49)	138 (40)
Rifampicin susceptibility found with additional diagnostics (%)	4 (7)	6 (9)	11 (15)	25 (25)	7 (15)	53 (15)
p-value for rifampicin susceptibility compared to all other levels (only tested patients)	0.002	0.0004	0.19	0.0001	0.66	-
No additional diagnostics for rifampicin resistance available (%)	12 (22)	13 (19)	42 (59)	67 (68)	17 (36)	151 (44)

Sputum smears for acid-fast bacilli collected at treatment initiation were available for 256 patients (75%). Eighty-five percent of patients who had a high bacillary load in the initial GeneXpert MTB/RIF sample had a positive sputum smear, compared to 74%, 21%, 24%, and 47% of patients with medium, low, very low, and not-reported bacillary loads, respectively (p<0.0001).

**Relation between false positive rifampicine resistance on GeneXpert MTB/RIF and patient outcomes:** patients who started MDR-TB treatment in 2019 had additional diagnostics for rifampicin resistance available significantly more often than patients who started in 2020 or 2021 (68% versus 49%, p=0.0006, Chi-square test). Of 53 patients who were found to have rifampicin-susceptible TB on follow-up diagnostics, 43 (81%) initiated treatment as an outpatient. Patient outcomes, stratified by the results of additional diagnostics for rifampicin resistance, are shown in Table 3. No patients were switched to standard TB treatment. In 19% of patients who were found to have rifampicin-susceptible TB with additional diagnostics, the treatment regimen was changed due to side effects (p=0.59 compared to other patients, Chi-square test). Patients in whom rifampicin-susceptible TB was found with additional diagnostics were cured at the end of TB treatment significantly more often compared to other patients (p=0.03, Chi-square test).

**Table 3:** outcomes of multidrug-resistant tuberculosis treatment, stratified by outcome of additional diagnostics for rifampicin resistance

Outcome	Rifampicin resistance confirmed (N=138)	Rifampicin susceptibility found (N=53)	No additional diagnostics available (N=151)	Total (N=342)
Changed regimen due to side effects (%)	28 (20)	10 (19)	36 (24)	74 (22)
**Final outcome**				
Cured (%)	68 (49)	34 (64)	71 (47)	173 (51)
Treatment failure (%)	4 (3)	1 (2)	3 (2)	8 (2)
Died (%)	4 (3)	0 (0)	8 (5)	12 (4)
Lost to follow up (%)	37 (27)	11 (21)	32 (21)	80 (23)
Still followed up (%)	25 (18)	7 (13)	37 (25)	69 (20)

## Discussion

Almost all patients in the MDR-TB program in Lusaka, Zambia, from 2019-2021 were initiated on MDR-TB treatment based on rifampicin resistance, as detected by GeneXpert MTB/RIF. This study shows that confirmatory diagnostic tests for rifampicin resistance, which were available in 56% of 342 patients, revealed rifampicin-susceptible TB in 28% of patients. Rifampicin-susceptible TB was detected significantly more often in patients who had very low bacillary loads on the initial GeneXpert MTB/RIF test. Seventy-eight percent of the patients with very low bacillary loads on the initial GeneXpert MTB/RIF test (in whom confirmatory diagnostic tests for rifampicin resistance were done; i.e. 25% of all patients with very low bacillary loads) had rifampicin-susceptible TB on confirmatory diagnostics.

This finding is in line with findings from other studies in sub-Saharan Africa, although the precise proportion of rifampicin-susceptible patients differs between studies. A previous, small-scale study from Zambia found that 14.3% of 21 samples that were rifampicin-resistant at the first test were susceptible at the follow-up confirmatory test [10]. In a Ugandan study, a false positive rate for GeneXpert MTB/RIF rifampicin resistance of 10.4% was found in patients who were referred for treatment of rifampicin-resistant TB [5]. In a Rwandan nationwide cohort study, only 53% of patients with rifampicin resistance detected by the initial GeneXpert MTB/RIF had confirmed rifampicin resistance, and a very low bacillary load was associated with false rifampicin resistance [4]. Studies from other parts of the world also found discordant results between GeneXpert MTB/RIF and phenotypic DST to be especially prevalent in samples with a very low bacillary load [11-15]. Similar problems have been reported with the GeneXpert MTB/RIF Ultra assay [16].

The mechanism behind false positive results for rifampicin resistance cannot be derived from our study. However, in a Chinese cohort, where 16.2% of patients who had rifampicin resistance according to GeneXpert MTB/RIF showed rifampicin susceptibility on phenotypic DST, Sanger sequencing revealed mutations in rpoB in 82.7% of these isolates, but this included a significant proportion of ??disputed´ mutations, which only confer a low level of rifampicin resistance [17]. Interestingly, most patients in whom no rpoB mutations were found had very low bacillary loads, and no rpoB mutations were found in most patients (68%) with discordant GeneXpert MTB/RIF and phenotypic DST results who had very low bacillary loads [17]. False positive results in this group might be related to GeneXpert probe delay and detection of resistance with probe B, as opposed to probe E [18,19]. Unequal efficacy of probe binding to diverse target sequences may be more pronounced in samples with a lower bacterial load [17]. Contrarily, false positive rifampicin resistance can also be found in samples with high bacillary loads, in which case it seems to be caused by unequal hybridisation dynamics [20].

The common finding of false positive rifampicin resistance results from GeneXpert MTB/RIF, especially in patients with very low bacillary loads, would support awaiting the results of additional diagnostics for rifampicin resistance prior to initiation of MDR-TB treatment. When a repeat GeneXpert MTB/RIF reveals rifampicin-susceptible TB, the patient can be initiated on standard TB treatment, while awaiting results from LPA and/or phenotypic DST. This is in line with the recommendations made by Ngabonziza and colleagues and the World Health Organisation [4,21]. Repeating GeneXpert MTB/RIF yields new results fast, and most patients with rifampicin-susceptible TB on additional testing in our cohort also had negative rifampicin resistance results on the repeat GeneXpert MTB/RIF. In another study, results from LPA and phenotypic DST were also mostly concordant with the repeat GeneXpert MTB/RIF test [11]. In addition, most patients in our cohort were able to receive outpatient treatment and did not need acute treatment for TB. Results from confirmatory diagnostics can therefore be safely awaited for most of the patients. Treatment outcomes for patients with rifampicin-susceptible TB in this cohort were favourable, which would further support that these patients can safely receive standard TB treatment when a repeat GeneXpert MTB/RIF reveals rifampicin susceptibility, while results from LPA and/or phenotypic DST are awaited. Furthermore, three-quarters of patients with very low bacillary loads on the initial GeneXpert MTB/RIF had smear-negative TB, and are likely not very contagious [22]. Recommendations on what to do while awaiting confirmatory results might depend on the local prevalence of MDR-TB, as well as that of very low bacillary load in samples with discordant rifampicin resistance results [21,23].

A caveat is that results from TB diagnostics are known to be variable. This can be related to variance in sample collection techniques and quality, but also due to the fact that TB cultures and phenotypic DST are imperfect golden standards [24]. Some cases where phenotypic DST reveals drug-susceptible TB might in fact be cases of MDR-TB, especially when the rifampicin minimal inhibitory concentration on phenotypic DST is close to the cut-off value that is used to determine resistance [25]. Furthermore, concurrent infection with drug-resistant and drug-susceptible strains is known to exist and can lead to conflicting results for drug-susceptibility testing. However, the magnitude of this phenomenon is not well-known [26,27]. This illustrates that MDR-TB diagnosis and treatment are complex, and can involve the consideration of conflicting drug-susceptibility results and clinical features in order to come to a treatment decision [28]. Based on our findings and the available literature, we do think that the chance that rifampicin resistance detected by GeneXpert MTB/RIF on a very low bacillary load sample in a setting with a low MDR-TB burden is quite likely to be a false positive result. When such a patient is not acutely ill, confirmatory diagnostics can be safely awaited.

Strengths of the present study include that this is an evaluation of an MDR-TB program in the real world over multiple years. Limitations are partially related to the patient selection. Only patients with pulmonary TB were included, and findings cannot be generalized to patients with extrapulmonary TB. Furthermore, because the cohort was selected from patients who were referred for MDR-TB treatment, patients in whom repeat GeneXpert testing was done at a local facility and did not confirm rifampicin resistance have not been included. Furthermore, we were unable to locate all files for patients initiated in the study period. Other limitations include missing data, especially on mycobacterial cultures, phenotypic DST, and LPA for some patients. This might be partially related to the coronavirus disease 19 (COVID-19) pandemic, as more data were missing for 2020 and 2021 compared to 2019.

## Conclusion

In patients treated for MDR-TB in Lusaka, Zambia, false positive rifampicin resistance results from the initial GeneXpert MTB/RIF were common. Confirmatory diagnostics revealed rifampicin susceptibility in 28% of tested patients. Rifampicin susceptibility was especially common in patients with very low bacillary loads on the initial test, where this was found in 78% of tested patients. We recommend repeating GeneXpert MTB/RIF in patients with very low bacillary loads and rifampicin resistance in the initial test, and only initiating MDR-TB treatment if rifampicin resistance is confirmed.

### What is known about this topic

- GeneXpert MTB/RIF can give false positive results for rifampicin resistance;
- This seems to be especially prevalent in the case of very low bacillary loads as detected by the assay.

### What this study adds

- False positive rifampicin resistance detected by GeneXpert MTB/RIF is also found in Lusaka, Zambia, in approximately 28% of patients referred for MDR-TB treatment;
- This study evaluated a real-world population.
